# Fabrication of Solid State Nanopore in Thin Silicon Membrane Using Low Cost Multistep Chemical Etching

**DOI:** 10.3390/ma8115390

**Published:** 2015-11-03

**Authors:** Muhammad Shuja Khan, John Dalton Williams

**Affiliations:** Electrical and Computer Engineering Department, The University of Alabama in Huntsville, Huntsville, AL 35899, USA; msk0003@uah.edu

**Keywords:** solid state nanopore, silicon, electrochemical etching, HF, atomic force microscopy

## Abstract

Nanopore-based analysis is currently an area of great interest in many disciplines with the potential for exceptionally versatile applications in medicine. This work presents a novel step towards fabrication of a single solid-state nanopore (SSSN) in a thin silicon membrane. Silicon nanopores are realized using multistep processes on both sides of n-type silicon-on-insulator (SOI) <100> wafer with resistivity 1–4 Ω·cm. An electrochemical HF etch with low current density (0.47 mA/cm^2^) is employed to produce SSSN. Blue LED is considered to emit light in a narrow band region which facilitates the etching procedure in a unilateral direction. This helps in production of straight nanopores in n-type Si. Additionally, a variety of pore diameters are demonstrated using different HF concentrations. Atomic force microscopy is used to demonstrate the surface morphology of the fabricated pores in non-contact mode. Pore edges exhibit a pronounced rounded shape and can offer high stability to fluidic artificial lipid bilayer to study membrane proteins. Electrochemically-fabricated SSSN has excellent smoothness and potential applications in diagnostics and pharmaceutical research on transmembrane proteins and label free detection.

## 1. Introduction

Biological and solid-state nanopores represent the two major classes of nanopore technology. Biological nanopores have been used for many years in single molecule (event) detection of protein, polypeptide, RNA, DNA sequencing, and other biomolecules with the advantages of high-throughput [1,2,3,4,5]. Unfortunately, these nanopores have limitations, such as the inability to modify the inner diameter of a protein nanopore and exhibit low stability with attached lipid bilayer membranes [2,6]. Among many protein channels (MspA, α-Hemolysin, bacteriophage phi29 connector channel, ENaC, ompF, gramicidin, ubiquinone, and melittin), phi29 embedded in a lipid membrane exhibits robust electrophysiological properties and has been proved to be very sensitive to demonstrate detection at single-molecule level [2,7]. Very recently, the phi29 connector channel is reported to investigate the translocation of dsDNA [8], detection of a single colon cancer-specific antibody [2], EpCAM in serum [9], and precise sensing of single chemicals by generating an 12 SH ring lining the channel wall [10]. The same group further investigates the behavior of single-stranded nucleic acids (ssDNA or ssRNA) by removing an internal loop segment from gp 10 subunit of the phi 29 channel [11].

Recent advancements in nanofabrication techniques allow artificial solid-state nanopores to be fabricated in Si_3_N_4_, SiO_2_, Si, and alumina [2,6,12,13,14,15,16,17,18,19,20,21,22,23,24,25]. These nanopore devices uncover many advantages, such as the ability to control a pore diameter, increased mechanical strength, and integration with micro/nano-devices. The use of single solid-state nanopores (SSSN) facilitates the monitoring and characterization of biomolecule species such as RNA, DNA, polypeptides, and other macromolecules at single molecular precision [2]. These advantages result increased stability of fused lipid membranes over a larger range of conditions such as ionic concentration, pH, and temperature and solid-state nanopore technology are currently being used to improve the detection of ion transport through protein channels [2,6,18,19,23,25].

To date, inductively-coupled plasma (ICP)-enhanced reactive ion etching (RIE) [13,17,21,22], focused ion beam (FIB) micromachining via transmission electron microscopy (TEM) technology [6,13,14,17,20,22,25], and electron beam lithography [2,12,18,20] have been used frequently to pattern micro and nanopores directly on the substrate after some modification. Despite the availability of large varieties in pore fabrication techniques, it is still very desirable to develop simpler and faster methods without using the above mentioned high energy electron beam techniques. Micro and nanopore production under chemically-processed environment do not only give freedom from using these high cost electron beam tools but also provide a controlled environment over pore geometry by electrochemical means at low current density.

Among many materials available, electrochemically-etched Si (or porous silicon) is a preferred support because of its biocompatibility and biodegradability [26]. These advanced properties allow silicon to be used as support structure for membrane proteins. One of the major advantages of micro/nanopores in Si is to use them in fabricating micro/nanoparticles and study their interaction with targeted tissues or pathogenic bacteria at cellular and molecular levels, as *in vivo* [26]. Larger-sized porous Si (PSi)-based microparticles also allow the loading of much larger quantities of drug and are appropriate for long-term (>4 month) therapies [27]. Silicon-based hybrid nanoparticles could also be beneficial in early detection and effective treatment of cancer [28].

Porosity of porous silicon membrane (n-type and p-type) is highly dependent on resistivity, concentration of HF in an aqueous electrolyte, and applied current density. Halder *et al.*, used dimethylformide (DMF) in HF to produce macropores with a current density of 3.5 mA/cm^2^ [29]. They performed electrochemically etching of heavily doped <100> p-type silicon membranes with a resistivity of 3 Ω·cm. In this case, average pore length does not change significantly even after 60 min and the resulting pores are not completely etched. Pore opening and depth are also realized with addition of acetonitrile (ACN) or cetyltri-methylammonium chloride (CTAC) in HF. A uniform pore size of approximately 1–2 µm in Si (p-type) with resistivity of 9–13 Ω·cm and 15.5 µm pore size in Si (n-type) with resistivity of 20 Ω·cm were produced by adding ACN and CTAC in an aqueous electrolyte, respectively [30,31]. To conclude, pores fabricated using DMF [22], CAN [23], and CTAC [24] are not etched all the way down even after increasing the current density from 3.5 to 15.5 mA/cm^2^. Moreover, their porous structures are not compatible with any biological sensing applications. To increase the stability of porous layer (p-type) with resistivity 10–20 Ω·cm, Tomoko *et al.* [32] introduced different types of alcohol (MeOH, EtOH, and BuOH) in electrolyte by keeping the concentration of HF constant with current density 14 mA/cm^2^. Unfortunately, pores fabricated in their research are blocked on the other side and are not compatible for studying any kind of protein channel activity.

Tantawi *et al.* [33] introduced low resistivity porous silicon membranes (n-type) with pores of about 0.5–2 µm in diameter by applying current density in the range of 10–15 mA/cm^2^. The thickness of the fabricated porous membrane was 3 µm and applicable to study biological membranes. Very recently, Burham *et al.* [34] investigated the effect of the most commonly used alcohols: ethanol, methanol, and propanol mixed with HF, forming an aqueous electrolyte for electrochemical fabrication. They claimed a PSi membrane with thickness less than 1 µm in n-type (0–100 Ω·cm) and p-type (0–100 Ω·cm) substrates with a current density of 25 mA/cm^2^. They reported the worst and stable pore formation by adding propanol and ethanol in HF, respectively. Results presented by Tantawi *et al.* [33] and Burhan *et al.* [34] are in support for investigating membrane proteins fused in lipid bilayers but their pores in porous membranes have irregular branches and variations in terms of pore size. These issues conclude that the sensing mechanism of a biological species might be unreliable in real-time measurements. Moreover, such porous membranes with varying pore size are not compatible for single molecule detection schemes and a large surface area of PSi may not be an acceptable platform to study protein translocation, direct DNA sequencing, virus detection, filtration of cancer cells, and single ion channel recording at the molecular level. To compensate for pore size and the need to reduce the number of ion mobility sites, a single solid state pore is required. Major applications include isolation of circular tumor cells [35], release of cancer cells with aptamer-functionalized micropores [36], and to provide a stable support to artificial lipid membranes to investigate transmembrane proteins [25].

In this research, we report a new method to fabricate a single solid-state nanopore (SSSN) of diameter 180 ± 12 nm in a thin silicon membrane (n-type) with resistivity of 1–4 Ω·cm. An electrochemical etching is performed for about 22 min with current density of 0.47 mA/cm^2^ in conjunction with low concentration of HF (5%) in an aqueous electrolyte without adding ACN, DMF, and CTAC. Moreover, simple ethanol (95%) is used instead of any other types, such as BuOH and EtOH. A small sensitive v-shaped groove cavity is fabricated in silicon (n-side) followed by photoassisted electrochemical etching. A narrow band blue LED is engaged to illuminate the silicon substrate. The dimension of an isolated pore is dependent on wafer resistivity, low current density, HF composition in electrolyte, and the etching time. Atomic force microscopy is further used to demonstrate the surface topography of fabricated pores.

## 2. Results and Discussion

**Figure 1:**
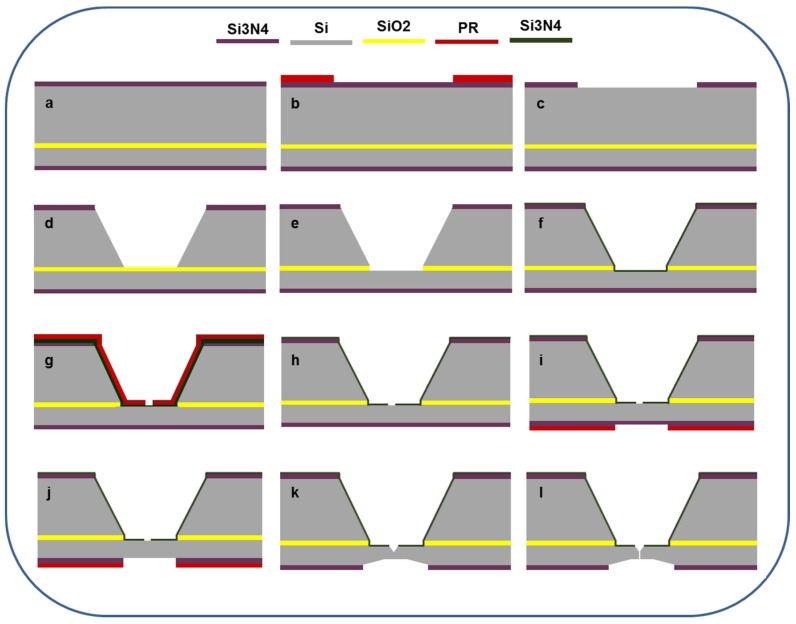
Cross-sectional schematic flow for the production of a single solid-state nanopore. (**a**) Deposition of 2 µm Si_3_N_4_ on both side of SOI wafer using PECVD; (**b**) patterning photoresist on backside of SOI wafer; (**c**) removal of exposed Si_3_N_4_ using RIE; (**d**) anisotropically etched Si using 30% KOH; (**e**) removal of SiO_2_ using 5 wt % HF; (**f**) sputtered 200 nm Si_3_N_4_ on Si (n-type); (**g**) patterning photoresist on n-type; (**h**) removal of exposed Si_3_N_4_ using RIE; (**i**) patterning photoresist on front side of the SOI wafer; (**j**) removal of exposed Si_3_N_4_ using RIE; (**k**) formation of v-shaped groove cavity on front side and square cavity on rear side of Si (n-type) using a low concentration of KOH; and (**l**) formation of a nanopore in thin silicon membrane using an electrochemical setup.

**Figure 2:**
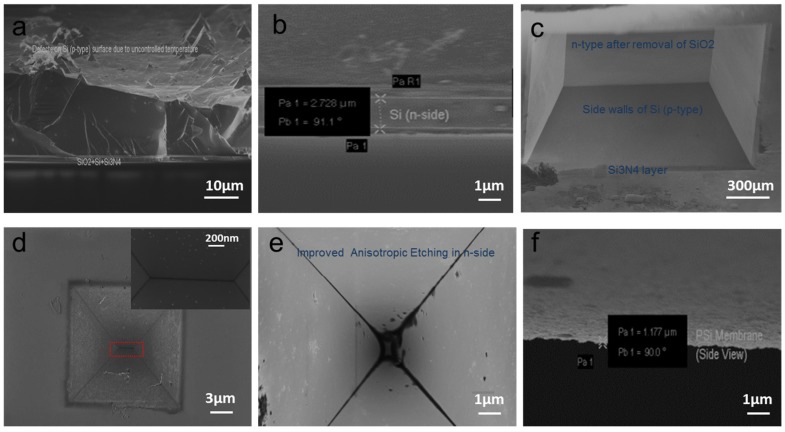
Formation of square and v-shaped groove cavities in silicon. (**a**) Small pyramids produced in the square cavity of Si (p-side) due to uncontrolled KOH concentration and temperature; (**b**) smooth favorable surface Si (p-side) with controlled KOH concentration and temperature; (**c**) Si (n-side) after removal of SiO_2_; (**d**) uneven pyramid with long v-shaped groove in Si (n-side); (**e**) controlled v-shaped small pyramid in Si (front side of n-type); and (**f**) thin Si membrane with thickness of 1.17 µm. (**a**,**b**,**f**) Cross sectional view; (**c**–**e**) Top view.

A detailed procedure and important parameters involved in fabrication of a single solid-state nanopore in a thin Si membrane are described in the experimental section. For brevity, as shown in Figure 1, a silicon nitride (Si_3_N_4_) layer with thickness 2 µm was deposited on both sides of the SOI wafer using plasma-enhanced chemical vapor deposition (PECVD) process. The back side of the SOI wafer was lithographically patterned and the exposed area of Si_3_N_4_ was then etched using reactive ion etching (RIE). Square cavity in p-type Si was achieved using 70% KOH at 65 °C. Small unwanted pyramids were observed in cavity after the completion of anisotropic etching. These could be due to high temperatures inside the cavities near the boundary walls of the bottom surface. It was difficult to see these pyramids from the top; therefore, a side view is presented in Figure 2a. To remove these pyramids and produce a favorable smooth surface, the substrate was immersed in 30% KOH at 55 °C for 7 h followed by four days at room temperature and the solution was changed every 24 h. The complete etched cavity with a smooth surface is shown in Figure 2b. The SiO_2_ layer was removed by dipping the SOI wafer in 5 wt % HF for 2 min and a smooth favorable surface of Si (n-type) is shown in Figure 2c.

Fabrication of v-shaped groove cavities on the front side Si (n-type) required special attention. As depicted in Figure 2d, despite all precautionary procedures, the inverted pyramidal structure was stopped at the center with long v-shaped groove dimension of 1.8 µm × 78 nm. Such a long shaped groove was not favorable to produce a single pore with high precision. This artifact is due to scattering of UV light on the edge of the mask and high KOH concentration. The most probable cause for the error is the misalignment of the <100> flat. Etching on the exact crystallographic axis using a quickly cut silicon flat is always prone to some angular error which results in a rectangular shape. Figure 2e shows the improved inverted pyramid which was finally obtained by dipping the sample in 3% KOH at 20 °C for 15 min. The effective thickness of the thin silicon membrane is 1.17 ± 0.05 µm as shown in Figure 2f.

To fabricate pores (nano/micro) in the thin silicon membrane, H_2_O and dimethylformide (DMF)-based HF are the two important commonly-electrolytic solutions used in electrochemical etching. The etching rate for DMF-based 5% HF is high, compared to H_2_O-based 5% HF [29]. In this work, an aqueous electrolyte is composed of HF (49%), ethanol (95%), and DI water. Since the membrane thickness is about 1.17 µm, concentration of HF used in this experiment is low. Three different electrolytes with concentrations of 15:40:45, 10:45:45, and 5:50:45 are studied to optimize the formation of pores in thin Si membrane.

**Figure 3:**
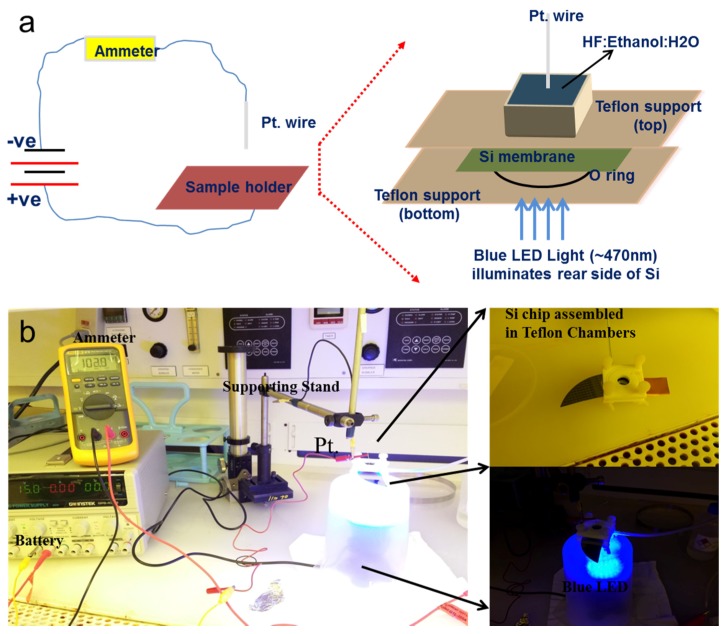
Photoassisted electrochemical fabrication of a solid-state nanopore. (**a**) Schematic description; (**b**) Experimental demonstration.

Figure 3 depicts the electrochemical HF setup which used a blue LED light to facilitate majority carriers (holes) in vertical direction in thin Si membrane. Detail of this method is described in Section 3.2 and Section 3.3. Three different pores are electrochemically fabricated with an average diameter of about 1.53 ± 0.11 µm, 520 ± 35 nm, and 180 ± 12 nm at the center of the inverted pyramid of the Si (n-type) using different concentrations of HF in an aqueous electrolyte. Current density is directly related to the pore size and low current density does not affect the side walls of the nanopore.

**Figure 4:**
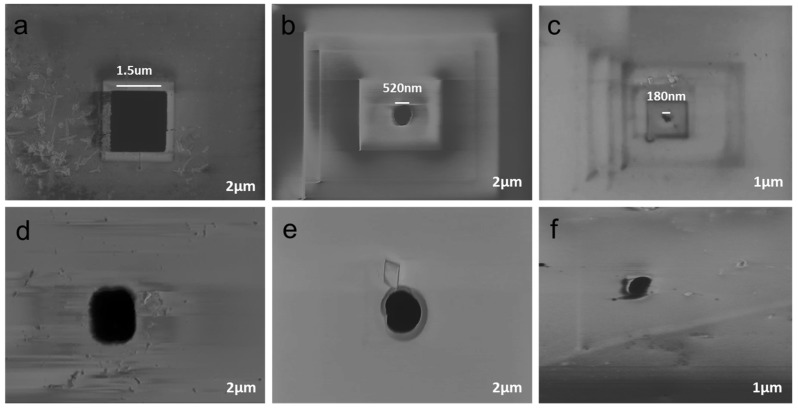
SEM images of fabricated pores in a thin Si membrane. (**a**–**c**) Front view; and (**d**–**f**) rear view. (**a**,**d**) 1.53 ± 0.11 µm under *J* = 4.91 mA/cm^2^ with 15% HF in an aqueous electrolyte; (**b**,**e**) 520 ± 35 nm under *J* = 1.57 mA/cm^2^ with 10% HF in an aqueous electrolyte; and (**c**,**f**) 180 ± 12 nm under 0.47 mA/cm^2^ with 5% HF in an aqueous electrolyte.

Optimized values are found by performing numerous experiments under different conditions. A choice of three different electrolytes is reported in this study to demonstrate the fabrication of single-solid state micro/nanopores. Depending on the desired pore geometry, the etching procedure was terminated immediately at a certain current level and the Si chip was immersed in ethanol (95%) for at least 30 min to minimize the effect of capillary action. The pore opening diameter was estimated using scanning electron microscopy (SEM) as revealed in Figure 4. Figure 4a–c depicts the front view pores electrochemically fabricated using 15:40:45, 10:45:45, and 5:50:45 electrolytes, respectively. Figure 4d–f show the rear view of pores.

**Figure 5:**
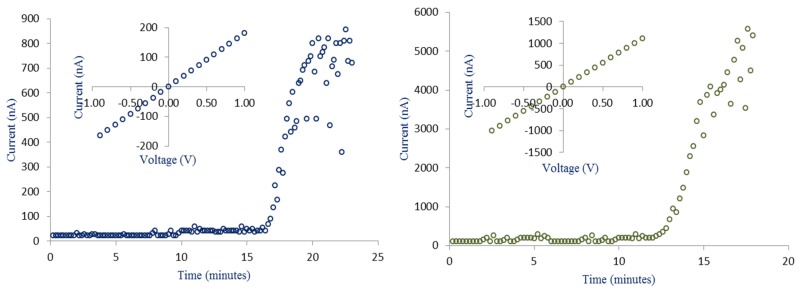
The electric current recorded during electrochemical etching as a function of time. Inset shows IV characteristic obtained in 7 wt % NaCl electrolyte. (**a**) 180 ± 12 nm produced in electrolyte having 5% HF concentration; and (**b**) 520 ± 35 nm produced in electrolyte having 10% HF concentration.

Figure 5 illustrates the current etching profile as a function of time and I-V characteristics of ~180 nm and ~520 nm solid-state pores. Figure 5a shows that pores of smaller size (~180 nm) take more time to fabricate as compared to the larger ones (Figure 5b). One of the main reasons is less concentration of HF (5%) used in the electrolyte. On the other hand, by increasing the HF concentration from 5% to 10%, lesser time is required to produce a ~520 nm pore size as illustrated in Figure 5b. When HF starts etching through the tip of the inverted pyramid, the process begins with creating a tiny pore. Initially, current remains constant and increases sharply at the time of the pore formation procedure. Depending on the desired pore size, one can terminate the etching procedure. This predicts that the diameter of a single nanopore can also be reduced to the sub-nanometer by controlling the etching time and using low HF concentrations in the range 0.5%–2.0%. Inset of Figure 5a,b show the I–V curves after pore opening.

A nanopore chip was sandwiched between two Teflon chambers filled with 7 wt % NaCl electrolyte. Pt electrodes were used as working and counter electrodes. High current indicates low resistance of the pore and *vice versa*. Ionic resistance of 5.494 ± 0.11 MΩ and 0.914 ± 0.14 MΩ are estimated for the fabricated pores of size 180 nm and 520 nm, respectively. These results are in close agreement with the resistance obtained after recording the stoppage current in the current-time profile (5.848 ± 0.13 MΩ and 0.892 ± 0.11 kΩ for 180 nm and 520 nm, respectively).

The experimental pore resistance is further compared with theoretical results using the given equation [37] and the results are in close agreement.

(1) $$R_{P}=\frac{4L}{\sigma_{b}\pi d^{2}}+\frac{L}{\sigma_{s}\pi d}$$

where *Rp* is the total pore resistance, *L* is the length, *d* is the diameter of the fabricated pore, and σ*_b_* and σ*_s_* represent the bulk and specific conductivity (7 wt % NaCl) of the electrolyte that fills the nano-channel. Using Equation (1), the resistance of the nanopore with absolute diameters 180 nm and 520 nm is 5.942 MΩ and 0.968 kΩ, respectively.

Table 1 summarized the important parameters involved in electrochemical fabrication. Total time is recorded for the completion of each experiment run at 5 V. The etch rate is consistent with recorded current density and is directly related with the bombardment of fluoride ions on the pore opening area. The final etch rate is obtained after the formation of a pore all the way down with a thickness of about 1.17 µm. Low concentrations of HF require more time to etch with an advantage of producing a narrow pore with diameter 180 ± 12 nm with current density 0.47 mA/cm^2^. The resistivity of n-type Si used in this study is 1–4 Ω·cm. Table 1 also describes information of other pores (~1.53 nm and ~520 nm) fabricated under different HF concentrations in this work.

**Table 1:** Summarized description of important parameters contributed to the fabrication of a single pore in a Si membrane. Thickness of each pore is about 1.17 ± 0.05 µm.

Pore Dimension	Electrolyte 49%HF:95%Ethanol:H_2_O	Current Density (mA/cm^2^)	Etch Rate (nm/min)	Estimated Time (min)
180 ± 12 nm	5:50:45	0.47	52.167	22
520 ± 35 nm	10:45:45	1.57	69.593	16
1.53 ± 0.11 µm	15:45:40	4.91	80.714	14

**Figure 6:**
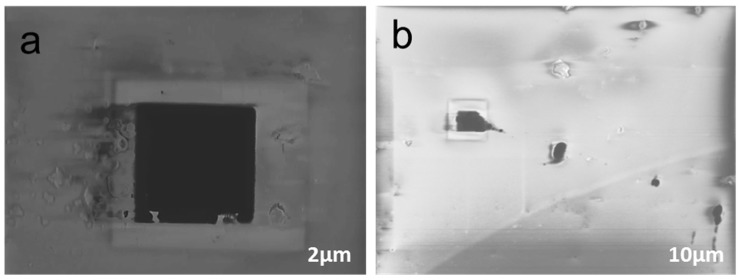
Effect of high concentration of HF (20%) in electrolyte with 15 V. (**a**) 70% cavity is etched under *J* = 8.6 mA/cm^2^; (**b**) micro/nanopores with size varying from 140 nm to 2.8 µm produced near the actual etched cavity.

Figure 6 depicts the effect of using 20% HF in electrolyte with an applied voltage of 15V. The etched area is dramatically increased to 5.1 µm with 70% of Si side walls are etched all the way down as shown in Figure 6a. Pore shape turns out to be a complete square as compared to the previous circular shape fabricated under 5V biasing with 5% HF. Some other pores with size in the range from 140 nm to 2.8 µm were also produced near the actual cavity as shown in Figure 6b. This could be due to the reaction of high concentrations of HF with Si_3_N_4_. These results are in support of fabrication of random nano/micropores in a thin silicon membrane to study filtration and trapping of cancer cells and other biomolecules at the cellular level. However, it is recommended that electrochemical etching procedures should always start at low biasing under low current density to fabricate an isolated nanopore of 100–200 nm in a thin Si membrane with resistivity of 1–4 Ω·cm.

### AFM Imaging

**Figure 7:**
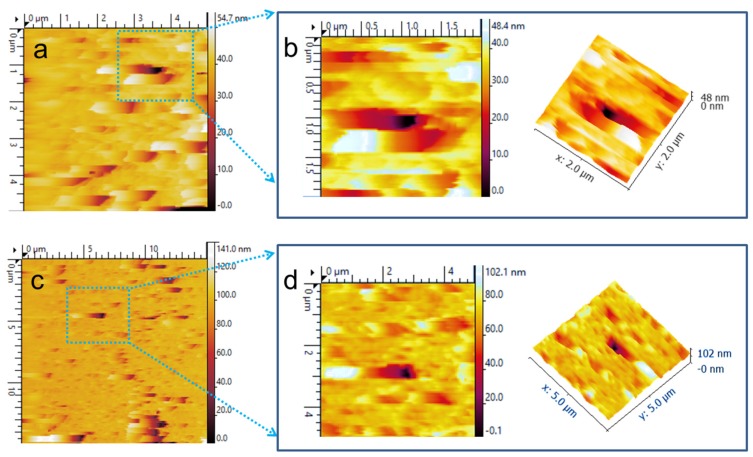
AFM imaging of pores fabricated in thin silicon membrane. (**a**,**b**) Sample A with pore size 180 ± 12 nm fabricated under *J* = 0.47 mA/cm^2^ with 5% HF and (**c**,**d**) Sample B with pore size 520 ± 22 nm fabricated under *J* = 1.57 mA/cm^2^ with 10% HF. (**a**) 5 × 5 µm^2^; (**b**) 2 × 2 µm^2^; (**c**) 15 × 15 µm^2^; and (**d**) 5 × 5 µm^2^.

To realize the surface morphology of pores using atomic force microscopy (AFM), we report two different samples (A and B) fabricated under different conditions. Sample A was fabricated in aqueous electrolyte with 5% HF and sample B with 10% HF. Figure 7a shows imaging with a scan size 5 × 5 µm^2^. A surface roughness value of the unprocessed surface is 0.78 ± 0.05 nm and is increased to 0.98 ± 0.07 nm after fabricating the thin silicon membrane using RIE and KOH procedures. To study the pore edges, AFM was performed at high resolution with a scan size 2 × 2 µm^2^. It can be seen that pore opening walls are almost straight all the way down instead of tilting towards the center. Moreover, pore edges exhibit a pronounced rounded shape as visible in Figure 7b. Such a type of smooth surface around he nanopore edges can provide high stability to fluidic lipid bilayer membranes.

For sample B, a large scan size with 15 × 15 µm^2^ was selected, as shown in Figure 7c, because of the large pore area fabricated using 10% HF in aqueous electrolyte. In this case, surface roughness is increased to 1.27 ± 0.08 nm which is almost 27% higher as compared to sample A. Another experiment was conducted with scan size 5 × 5 µm^2^ as shown in Figure 7d. Surface roughness for this scan size is slightly higher (1.08 ± 0.03 nm) than sample A with the same scan size (Figure 7a). It is noted that pore edges are not uniform with higher HF concentration in electrolyte. Results obtained using SEM and AFM are in agreement for the pores produced with 5% and 10% HF under current density 0.47 mA/cm^2^ and 1.57 mA/cm^2^ respectively.

## 3. Experimental Section

### 3.1. Fabrication Layout

Silicon-on-insulator (SOI) wafer <100> (purchased from Silicon Valley Microelecronics, Inc. (Santa Clara, CA, USA)) is used as a substrate with dimension: 500/2/2.5 ± 0.5 µm. Low stress silicon nitride (Si_3_N_4_) layer with thickness 2 μm was deposited on both sides of SOI wafer using plasma enhanced chemical vapor deposition (PECVD), process as shown in Figure 1a. An array of 1 × 1 mm^2^ squares was lithographically patterned (Figure 1b) on the back side of SOI wafer using SPR220 positive resist. As depicted in Figure 1c, RIE (790, Plasma-Therm, Saint Petersburg, FL, USA) was then used at 75 W with 30 mTorr pressure, CF_4_: 36 sccm and O_2_: 4 sccm by volume to etch the exposed area of Si_3_N_4_. Etch rate was determined using both a surface profiler (P10, KLA-Tencor, Milpitas, CA, USA) and a white light interferometer (WYKO NT1100, Veeco, Tucson, AZ, USA) after the removal of the photoresist mask. Square cavity of 200 × 200 µm^2^ in p-type Si (Figure 1d) was achieved using 30% KOH at 55 °C for 7 h followed by four days at room temperature with total etched thickness of 500 µm. Etching procedure was monitored using SEM(LEO 1550) every 12 h. SiO_2_ layer was then removed (Figure 1e) by dipping SOI wafer in 5 wt % HF for 2 min and then 15 min in ethanol.

A thin layer of Si_3_N_4_ (300 nm) was sputtered on Si (n-type) with deposition rate of 22.1 nm/min at low power of 100 W with pressure 5 mTorr (shown in Figure 1f) followed by an aligned contact lithography patterning and etching of square window 10 × 10 µm^2^ over the center of the membrane (shown in Figure 1g,h). Etching rate for sputtered Si_3_N_4_ layer (300 nm) was 9.98 nm/min at 50 W with 30 mTorr in conjunction with CF_4_ (30 sccm) and O_2_ (6 sccm).

Another square window of 500 × 500 µm^2^ was patterned and etched on the front of the SOI wafer (n-type Si) (shown in Figure 1i). Finally to fabricate the thin silicon membrane with thickness of about 1.17 µm (Figure 1k), chip was immersed in 10% KOH at 20 °C for 15 min. This critical step has already explained in results and discussion section with the help of SEM.

Any impurity can highly affect the nanopore formation. Therefore, substrate was immersed in 5 wt % HF for 30 s to remove any contamination. To fabricate an ohmic contact on front side of SOI wafer, a thin layer of aluminum (100 nm) was sputtered on n-type silicon. Aluminum contact pads were thermally annealed at 130 °C for about 60 min followed by 350 °C for 10 min. Copper foil was then arranged beneath the ohmic contact for use in anodic photoassisted chemical etching of the thin silicon membrane to produce SSSN.

### 3.2. Choice of Light and DC Biasing

To illuminate the back side of n-type Si, a halogen lamp is the most commonly used light source. It generates holes throughout the volumes of the etched silicon material which can start etching in lateral direction (very similar to p-type silicon) and heat the sample due to continuous infrared light. To overcome these issues, light intensity can easily be controlled either by adjusting the distance between the Si wafer under etching and the light source or using light emitting diodes (LED) as a light source instead of regular halogen lamps. Therefore, in this work, a blue LED was used to facilitate majority carriers in to fabricate straight pores in the thin silicon membrane.

Controlled DC biasing is one of the major contributions in deciding the pore opening diameter and its depth. It is recommended to start electrochemical etching always at low operating voltage. Since the area covered by the opening of the inverted pyramid is too small and an excessive bombardment of fluoride ions can cause immediate damage to the tip of the inverted pyramid in n-type Si, therefore, we recommend applied biasing must start at 0.5 V and then gradually increase to 5 V with an increment of about 1–10 mV/s. Among many experiments performed at this biasing condition, three important results are reported and summarized in this study.

### 3.3. Electrochemical HF Setup

The silicon membrane needs to be handled carefully because the thin membrane is easy to break during electrochemical etching procedure. Si device with multiple cavities was sandwiched between two Teflon supports using O-rings to prevent any leakage of electrolyte and to apply low stress on thin Si membrane. All screws were tightened gently to avoid any cracking on the mounted chip. This setup was also tested by pouring ethanol in the cell for at least 45 min. The assembled system was then placed in the designated fume hood for handling HF. The Si square cavity on the backside of the SOI wafer was filled up with an aqueous electrolyte (49% HF, 95% ethanol and H_2_O). Platinum (Pt) wire was immersed in the electrolyte and the distance between the Pt wire and pore opening area in the inverted pyramid was about 1 mm. The etching cell was connected in series with an ammeter to monitor and record the current passing through the cell. Potentiostatic control is applied to the electrochemical etching setup. Recorded current is not the absolute value but it is the current density in mA/cm^2^. Current density is recorded after completing the etching procedure of the thin Si membrane and is finalized after careful consideration of the area covered by the pore.

### 3.4. AFM

Characterization of the fabricated nanopore was performed using atomic force microscopy (Pico plus AFM 1550 instrument from Molecular Imaging, (Keysight Technologies, Santa Rosa, CA, USA) in non-contact mode. In order to remove any contamination on the sample’s surface, the sample was sonicated for 15 min and dried using a nitrogen spray gun. AFM results were demonstrated with scan sizes (15 × 15 µm^2^, 5 × 5 µm^2^ and 2 × 2 µm^2^) to reveal the fabricated nanopore at high resolution. A cantilever with a spring constant of 10 N/m (purchased from Bruker Inc. Camarillo, CA, USA) was used for all imaging. Two different samples (A and B) fabricated under different conditions were presented in the results and discussion sections.

## 4. Conclusions

A single solid-state nanopore (SSSN) is fabricated in this work without using electron beam facilities. Silicon is selected as the preferred material due to its biodegradability. In this contribution, we present the detailed process to fabricate an isolated nanopore in a thin silicon membrane using facile, inexpensive, and reproducible multistep chemical etching at low concentrations of HF in electrolyte. The use of a blue LED is recommended to fabricate straight nanopores in n-type Si. Methodology discussed in this study relies only on wet chemicals for silicon etching, with no requirement of specialized ion-beam and electron beam facilities, and regardless of the initial silicon-wafer thickness. The electrochemical etching is performed in low doped n-type Si with resistivity 1–4 Ω·cm under potentiostatic control in the range from 0.5 V to 5 V. Nanopore 180 ± 12 nm is obtained at low current density of 0.47 mA/cm^2^ without using any organic solvent (DMF, can, and CTAC) and other types of alcohols (MeOH, EtOH, and BuOH) in an aqueous electrolyte. Single nanopore fabrication in n-type Si was challenged and this work counts as an additional step in solid-state nanopore research. Such single solid-state pores (micro and nano) in thin silicon membranes provide an alternative platform to perform electronic sensing of a single biomolecule at molecular and cellular levels. It could be an alternative platform to support artificial lipid membranes with long-term stability to investigate protein channel activity.
